# Magnitude, trajectory and determinants of antenatal depressive symptoms in Adama Town and East Shewa Zone, Ethiopia: evidence from a prospective longitudinal eCohort survey

**DOI:** 10.1136/bmjopen-2026-120181

**Published:** 2026-10-07

**Authors:** Gebeyaw Molla Wondim, Wubegzier Mekonnen, Catherine Arsenault, Anagaw Derseh Mebratie, Margaret E Kruk, Damen Haile Mariam

**Affiliations:** 1 Health System and Reproductive Health Research Directorate, Ethiopian Public Health Institute, Addis Ababa, Ethiopia; 2 School of Public Health, Addis Ababa University College of Health Sciences, Addis Ababa, Ethiopia; 3 Department of Reproductive, Family and Population Health, School of Public Health, Addis Ababa University, Addis Ababa, Ethiopia; 4 Department of Global Health, The George Washington University, Washington, District of Columbia, USA; 5 School of Medicine, Washington University in St Louis School of Medicine, St. Louis, Missouri, USA; 6 School of Public Health, Addis Ababa University, Addis Ababa, Ethiopia

**Keywords:** Depression & mood disorders, Pregnant Women, Longitudinal studies, Ethiopia, Antenatal, Prenatal diagnosis

## Abstract

**Abstract:**

**Objectives:**

Antenatal depression is a significant yet under-recognised global public health concern that affects a substantial proportion of women in Ethiopia. This study aimed to examine the magnitude, trajectory and determinants of depressive symptoms among pregnant women.

**Design:**

A prospective longitudinal study design was conducted between April 2023 and February 2024.

**Setting and participants:**

The study was conducted in Adama Town and the East Shewa Zone of the Oromia Region, Ethiopia. A total of 933 pregnant women who visited the health facility for their first ANC visit were recruited and followed throughout their pregnancy.

**Primary and secondary outcome measures:**

Antenatal depressive symptoms were measured using the Patient Health Questionnaire-9 through face-to-face interviews and monthly phone calls. The trajectory and determinants of antenatal depressive symptoms were assessed using group-based trajectory modelling and generalised estimating equations.

**Results:**

The study found that overall, 30.5% of participants experienced antenatal depressive symptoms, with a lower prevalence at the first measurement point (24.2%) and higher prevalence at the second measurement point (36.9%). Antenatal depressive symptoms were higher during the first trimester (35.6%) compared with the second (19.8%) and third (13.2%) at baseline, and the pattern was consistent across the monthly follow-up period. Moreover, 11.6% of pregnant women had a trajectory of mild-to-moderate depressive symptoms over time. Factors included being currently married (adjusted OR (aOR) 0.53; 95% CI 0.28 to 0.98), having an unintended pregnancy (aOR 1.29; 95% CI 1.03 to 1.62), experiencing intimate partner violence (aOR 2.43; 95% CI 1.67 to 3.56), experiencing at least one pregnancy danger sign (aOR 2.60; 95% CI 2.22 to 3.04), having a history of at least one pregnancy complication (aOR 1.87; 95% CI 1.53 to 2.29), being in the first trimester (aOR 1.45; 95% CI 1.09 to 1.93), having poorer self-reported health (aOR 1.66; 95% CI 1.40 to 1.97) and moderate to low health-related quality of life (aOR 2.43; 95% CI 1.78 to 3.32) were associated with antenatal depressive symptoms.

**Conclusion:**

Nearly one-third of pregnant women had antenatal depressive symptoms, and one in 10 demonstrated steadily increased depressive symptoms over time. The high burden of antenatal depressive symptoms and their association with preventable social and pregnancy-related factors underscore the need for comprehensive antenatal care services that incorporate mental health screening, psychosocial support and interventions addressing intimate partner violence and pregnancy-related complications.

**Strengths and limitations of the study**

- The study employed a prospective longitudinal study design, evaluated through advanced statistical methods, specifically generalised estimating equations and group-based trajectory modelling, to track antenatal depressive symptoms over time.
- We employed a locally validated tool to accurately measure antenatal depressive symptoms over time.
- The participants’ selection from a health facility and the presence of attribution bias could limit the generalisability of the findings.
- The responses were based on the participants’ self-reported data, which introduces potential social desirability and recall bias for capturing sensitive mental health and pregnancy experiences.
- Lastly, using a phone survey for follow-up data collection may have led to underestimation or overestimation of symptom severity, while failure to assess women’s stressful events and COVID-19 status further limits the findings.

## Introduction

 Pregnancy is a major life event for women, involving various physiological and psychological changes.^1^ Depression is characterised by different symptoms such as feelings of sadness, loss of interest or pleasure in activities, sleep disturbances, difficulty concentrating or suicidal thoughts.^2 3^ These symptoms can, in turn, impair the functioning of maternal body systems by disrupting the glucocorticoid hormonal balance.^4 5^ Depression increases the risk of obstetric complications such as hyperemesis, miscarriage, pre-eclampsia and labour problems^6 7^ leading to poor birth outcomes and postpartum depression.^8–11^ Moreover, it negatively affects women’s health-seeking behaviour^12 13^ and increases the risk of maternal maltreatment of offspring.^14–16^

Depression accounts for 6.2% of years lived with disability during pregnancy,^17–19^ and it is a significant public health concern affecting 34% of pregnant women in low-income and middle-income countries.^20^ In Ethiopia, different observational studies have shown that antenatal depressive symptoms affect 6.9% to 47.6% of women^21 22^ and the pooled prevalence of those studies in Ethiopia accounted for 24.6%.^23^ Despite this burden, only 2.2% of women in low-income and middle-income countries (LMICs) received the minimum adequate treatment for antenatal depressive symptoms including psychological or medical treatment.^24 25^

Despite inconsistent findings in previous studies conducted in LMICs,^9 20 26^ including Ethiopia,^7 21 22 27–32^ these studies relied on a single time-point measurement of depressive symptoms during pregnancy. However, as the antenatal care (ANC) guidelines have shown, pregnant women should be screened for depressive symptoms at each trimester, and women who screen positive should be referred for further investigation and psychosocial support.^33^ Consequently, studies have shown a notable measurement gap for determining the burden and examining the trajectory of antenatal depressive symptoms over time.

Furthermore, the existing studies have identified several factors associated with antenatal depressive symptoms, including sociodemographic-related problems,^34–37^ pregnancy-related problems (such as obstetric complications, history of abortion and unplanned pregnancy),^34–38^ psychosocial stressors (such as poor social support and intimate partner violence)^34 35^ and maternal lifestyle (ie, substance use) during pregnancy.^31 39^ However, critical determinants such as women’s self-rated health status, health-related quality of life, maternal anaemia and malnutrition have not been adequately examined.

A longitudinal study is one of the most powerful observational designs for tracking real-time health changes over time, rather than snapshot measurements. Therefore, longitudinal assessment of antenatal depressive symptoms is crucial to understand the burden and trajectories of symptoms over time. Evidence from such studies is vital for policymakers when developing or updating clinical guidelines, as well as for healthcare practitioners designing targeted screening and treatment cascades for depression. Consequently, this study aimed to address the previously mentioned gaps and to assess antenatal depressive symptoms longitudinally to determine the magnitude, trajectory and predictors.

## Methods

### Study design, setting and period

This study used data from the eCohort study, a prospective longitudinal study design conducted among pregnant women to obtain real-time data on women’s experiences and health system quality throughout the continuum of care. The study was conducted in Adama Town and the East Shewa Zone, from April 2023 to January 2024. Adama Town and the East Shewa Zone are located in the Oromia Regional State, Ethiopia. Adama Town is the most densely populated city in the Oromia Region, while East Shewa Zone is the second-largest zone in the region, comprising 13 woredas. There are 126 public and private healthcare facilities in Adama Town and 103 in the East Shewa Zone, offering healthcare services. Further details on the study methods have been published elsewhere.^40^

### Study population and eligibility criteria

Regardless of the trimester level, all pregnant women who visited the selected health facilities in Adama Town and East Shewa Zone for their first ANC visit were contacted. A total of 21 public and private health facilities were selected, including hospitals, health centres, and specialised maternal and child health clinics. Consequently, pregnant women who fulfilled the following criteria were recruited from April to May 2023 at baseline: (1) pregnant women who visited the health facility for their first ANC visit; (2) who intend to continue their pregnancy; (3) those who intend to live in the study area, (3) those who had a personal phone number or volunteered to receive a phone from the survey for follow-up data collection purpose; (4) those who did not visit the health facility solely for pregnancy confirmation; (5) do not have hearing or communication problems (6); volunteered for audio recording of the follow-up data and (7) provided a signed informed consent.

### Sample size determination

The required minimum sample size of the study was determined using a single population proportion formula, considering the proportion of pregnant women who experienced depressive symptoms at least once (26.2%) in the south-western part of the Oromia Region,^41^ 95% CI, 4% margin of error, 1.5 design effect, 23.3% lost to follow-up^41^ and a 10% non-response rate at baseline. Following this, the required minimum sample size for follow-up was 928. To satisfy the minimum required sample size, we selected pregnant women who had at least two measurements of antenatal depressive symptoms and were followed up to the end of the pregnancy from the eCohort study. Consequently, a total of 933 pregnant women were included.

### Data collection process and instruments

Data were collected using a pretested and structured questionnaire. The survey instruments included sociodemographic, maternal, behavioural and psychosocial characteristics, as well as self-rated health status, health-related quality of life and pregnancy outcome measures. The instruments were administered at baseline and at monthly intervals during follow-up. The baseline data were collected through face-to-face interviews, while the follow-up data were collected through phone interviews using the established call centre. A cell phone with a Subscriber Identity Module (SIM) card was provided to participants for follow-up data collection purposes. Participants were contacted monthly, and three attempts were made daily for a week to contact them for an interview.

### Data quality control

The data quality assurance was based on the protocol of the eCohort longitudinal study.^40^ Face and content validity of the tools were assessed and reviewed by researchers and experts working in health facilities and academic institutions. The questionnaire was translated into two local languages: Amharic and Afaan Oromo. A 10-day training was given for data collectors and supervisors. A pilot test was conducted in Addis Ababa and Sheger City health facilities over 2 months. Data were collected exclusively by females, who fluently speak and write the local languages and have expertise in public health, nursing and midwifery. Supervision and spot-checking were conducted by supervisors and coordinators, with immediate feedback given as needed. Data were collected using REDCap software, and all phone call data were recorded and reviewed via audio transcription for quality and consistency checking. We assessed the internal validity of the current data across measurements using Cronbach’s alpha (α>0.77), which demonstrated good reliability.

### Study variables, measurements and operational definitions

#### Antenatal depressive symptoms

Antenatal depressive symptoms were measured using the self-reported Patient Health Questionnaire (PHQ-9), which consists of 9 items used to assess the intensity and frequency of depressive symptoms over the last 2 weeks. Each item has four Likert scale response options, ranging from 0 (not experienced at all) to 3 (experienced nearly every day). The total score of PHQ-9 ranges from 0 to 27 and is categorised into five levels of depressive symptoms, such as normal/minimal (0–4), mild (5–9), moderate (10–14), moderately severe (15–19) and severe.^20–27 42^ The tool was validated among pregnant women through the Afaan Oromo language with a sensitivity and specificity of 84% and 79.4%.^43^ We assessed women’s depressive symptoms every month until the end of pregnancy, and participants with a PHQ-9 score ≥5 at every measurement point were considered to have antenatal depressive symptoms.^7 44^

#### Sociodemographic characteristics

The sociodemographic-related variables included marital status (currently married and not currently married), education (no education/some primary, complete primary/some secondary, completed secondary/higher), occupation (employed, merchant, housewife, others), age (<20, 20–34 and ≥35 years), place of residence (urban, rural) and religion (Christian, Muslim and other) were assessed from participants’ self-report. The wealth index of the participant was created using household assets and the material construction of the house, using a similar method used by the Ethiopia demographic and health survery.^45^ We computed the wealth index score using PCA, and the scores were categorised into three tertiles: poorest, middle and richest.

The pregnancy and clinical variables included parity (one vs more than one), gravidity (primigravida vs multigravida), pregnancy intention (intended vs unintended), and having at least a history of obstetric complications (abortion, stillbirth, preterm birth, delivery complications, caesarean section (CS) delivery, prolonged labour, congenital anomalies and neonatal deaths), which were assessed through the mother’s self-report and reviewing the maternal health card. The experience of pregnancy danger signs, at least one of which included severe or persistent headache, vaginal bleeding, fever, severe abdominal pain, breathing difficulty, convulsions or seizures, loss of consciousness, blurred vision and complete cessation of fetal movement, was assessed through the mother’s self-reported ‘Yes/No’ responses,^33^ both at baseline and monthly.

#### Pregnancy, clinical, psychosocial and behavioural variables

Gestational age was determined at baseline using the self-reported date of the last menstrual period and the date of enrolment; accordingly, participants were categorised into trimester levels: first trimester (<13 weeks), second trimester (13–27 weeks) and third trimester (≥28 weeks). For the follow-up period, the interviews were conducted monthly until the end of pregnancy, and at each subsequent measurement point, gestational age was updated based on the elapsed time from the baseline interview date to the follow-up interview date and re-categorised into the corresponding trimester level in each follow-up.

We assessed the women’s haemoglobin level by conducting a haemoglobin test using HemoCue and by extracting data from the maternal health card. Pregnant women with a haemoglobin level <11 g/dL were considered to have anaemia.^46^ We assessed women’s nutritional status by measuring mid-upper arm circumference using a mid-upper arm circumference (MUAC) tape. Pregnant women with a MUAC less than 23 cm were considered malnourished.^47^ The overall health status of women was evaluated using a self-rated Likert-scale question, “How do you rate the current overall health status?”, and the responses were categorised as excellent, very good, good, fair and poor. We assessed women’s health-related quality of life using the self-reported EuroQol 5-Dimension 3-Level (EQ-5D-3L).^48 49^ We computed the total score of the EQ-5D-3L, considering Zimbabwe’s preference-based tariff score,^50^ which ranged from 0 to 1. The scores were categorised into low (<0.70), moderate (0.70–0.84) and high (≥0.85) health-related quality of life. Psychosocial and behavioural factors included having experienced any form of intimate partner violence during pregnancy,^33^ and the use of any of these substances, including alcohol, khat and cigarettes,^51^ were assessed through the mother’s self-reported Yes/No response.

#### Data management and analysis

Data cleaning and analysis were conducted in Excel and Stata version 18 software. We used a complete-case analysis, considering the assumption of missing completely at random. For PHQ-9 item missing values, we handled them by using person-mean imputation, replacing the missing item with the mean value of the participant’s completed items for that assessment time. Descriptive statistics, including frequency with percentage and mean with SD, were used. The proportion of antenatal depressive symptoms reported at each measurement point and the trajectory of antenatal depressive symptoms were assessed using group-based trajectory modelling (GBTM). Participants were assigned to the specific class membership based on antenatal depressive symptom scores measured up to six time points. We fitted 2 to 5 group models, considering a censored normal model with a quadratic polynomial order. The optimal model was selected based on the lowest Bayesian Information Criterion (BIC)/Akaike Information Criterion (AIC) values. Additionally, a minimum class sample size of >5%, an average posterior probability of each class exceeding 0.7, a model entropy value greater than 0.7 and the distinct interpretability of the trajectory plots^46 52^ were also considered.

We used a generalised estimating equation with a logit link function to assess the determinants of antenatal depressive symptoms, which accounted for repeated measurements of antenatal depressive symptoms^47^ and correlation among observations within a subject (cluster) by assuming a working correlation matrix.^53^ Antenatal depressive symptoms were categorised into a binary outcome at each measurement point. An exchangeable working correlation structure was used, as the within-subject correlation of antenatal depressive symptoms showed similar patterns over time, and the models had a lower quasi-likelihood information criterion (QIC) value compared with other alternative working structures (online supplemental table 1).

We performed attrition bias analysis to compare baseline characteristics and outcome measures between participants who completed all six time points and those who dropped out before. Differences were evaluated using the χ² and the standardised difference. Additionally, logistic regression analysis was conducted to examine whether baseline predictors and baseline outcome measures predicted the dropout status. Additionally, we conducted a competing risk analysis by categorising participants’ follow-up status into three groups: (1) those who completed follow-up (completers), (2) those who were lost to follow-up and (3) those who were lost to follow-up due to natural pregnancy completion (delivery/abortion/stillbirth) to determine whether the participants’ baseline characteristics predicted study attrition. We applied a Fine-Gray subdistribution hazard model to identify predictors of true lost follow-up, treating those who did not complete follow-up due to a natural pregnancy outcome as a competing event. All baseline variables with complete observations were included in the model, and variables with a p value <0.05 in the final model were considered risk factors for true lost follow-up. Furthermore, we performed a sensitivity analysis, restricted to those who completed all six rounds, to assess whether attrition bias influences the generalised estimating equation (GEE) estimates and the robustness of findings.

Multicollinearity among independent variables was checked using the variance inflation factor and a pairwise correlation matrix. The results showed a low risk of multicollinearity (variance inflation factor <0.3 and r<0.6). Bivariable and multivariable analyses were conducted to examine the determinants of antenatal depressive symptoms. In addition to variables with p values <0.2 in the bi-variable analysis, the theoretical relationship of variables with antenatal depressive symptoms was considered for selecting independent variables for multivariable analyses. The best-fit final model was selected based on the lowest value of the quasi-likelihood information criterion unbiasedness (QICu). Finally, variables with a p value <0.05 in the multivariable analysis were considered predictors of antenatal depressive symptoms, and the estimated population parameters were presented and interpreted as adjusted ORs (aORs) with 95% CIs.

We performed three sensitivity analyses: (1) restricting the analysis to women enrolled in their first trimester and followed until the end of pregnancy (up to six time points), (2) restricting to those who completed all four rounds regardless of trimester level and (3) considering a PHQ-9 ≥10 as a cut-off point for defining antenatal depressive symptoms to compare the robustness of the finding.

### Patients and public involvement

Patients were not involved in the design, implementation, reporting and dissemination of the study. However, stakeholders from academic institutions, the research institute and healthcare providers at health facilities participated in the study’s design and implementation and in the dissemination of findings.

### Ethical considerations

The eCohort study was approved by the Ethiopian Public Health Institute and the Harvard University TH Chan School of Public Health IRB, with reference numbers EPHI-448-2022 and #RB22-0487, respectively. Formal informed consent was obtained from all study participants, and all ethical considerations, including participants’ written informed consent, privacy and confidentiality, their right to withdraw at any time, and an explanation of risks and benefits, were assured.

## Results

### Characteristics of study participants

Table 1 presents the study flow from enrolment to endline. Of 933 pregnant women enrolled in the study, all were assessed in the second round, 854 (91.5%) were assessed in the third round, 728 (78.0%) in the fourth round and only 85 (9.1%) of women completed all seven rounds of measurement points. The primary reasons that participants did not complete all seven measurement points were: lost to follow-up (8.2%) and no longer eligible to assess antenatal depressive symptoms due to the end of pregnancy (82.4%). A total of 58 pregnant women had no antenatal depressive symptoms, with 22 pregnant women at six rounds, 18 at five rounds, 11 at the fourth round and only one pregnant woman at the third round (table 1). The average time interval between measurement points was 4.2 weeks (SD±0.61).

**Table 1:** Study flow of the participants across the seven time points among pregnant women in Adama town and East Shewa Zone, Ethiopia, 2025

Survey time	Eligible participants (n)	Absent (n)	Lost to follow-up (n)	Pregnancy ended (n)*	Participants assessed (n)	Cumulative retention (%)
Time 1	933	0	0	0	933	100.0
Time 2	933	0	0	0	933	100.0
Time 3	933	1	31	47	854	91.5
Time 4	855	11	17	99	728	78.0
Time 5	738	18	10	205	506	54.2
Time 6	524	22	11	251	240	25.7
Time 7	262	0	10	167	85	9.1
Total	5179	52	79	769	4279	59.6

* Including abortion, stillbirth and live births.

Table 2 presents the participants’ baseline characteristics; the mean age of the participants was 25 years (SD±4.68). Nearly all participants were currently married, and 289 (39.8%) had completed primary or some secondary level of education. Two-thirds (66%) of the participants were in their second trimester, 247 (26.5%) were primigravida and 683 (73.3%) reported their pregnancy was intended. Regarding women’s clinical characteristics, 94 (10.7%) were anaemic, 202 (21.7%) were malnourished and 617 (66.1%) experienced at least one pregnancy danger sign (table 2).

**Table 2:** Participants’ sociodemographic, pregnancy, obstetric and clinical characteristics, Adama Town and East Shewa Zone, Ethiopia, 2025 (n=933)

Characteristics	Frequency	Per cent (%)*
Sociodemographic		
Age in years, mean (SD)	25.4 (4.8)	
Residence		
Rural	475	50.9
Educational status		
No education or some primary	356	38.2
Complete primary or some secondary	373	40
Complete secondary or higher	204	21.9
Currently married	911	97.6
Occupation		
Employed	176	18.9
Merchant	139	14.9
Housewife	546	58.5
Others†	72	7.7
Religion		
Christian	659	70.6
Muslim	244	26.2
Others	30	3.2
Economic status (n=915)		
Poorest	304	33.2
Middle	304	33.2
Richest	307	33.6
Socio-behavioural factors		
Experienced intimate partner violence	59	6.3
Substance use‡	227	24.3
Pregnancy and obstetric characteristics		
Primigravida	247	26.5
Intended pregnancy	683	73.3
Gestational age at enrolment		
First trimester	275	29.9
Second trimester	607	66.0
Third trimester	38	4.1
History of obstetric complications§	303	32.5
Experienced pregnancy danger signs¶	316	33.9
Experienced pregnancy complications**	141	15.1
Medical characteristics		
Women with chronic illness††	100	10.7
Anaemia (Hgb <11 g/L) (n=881)	94	10.7
Malnourished (MUAC <23 cm)	202	21.7
Self-rated health status		
Excellent/very good	346	37.1
Health-related quality of life		
Moderate/low	78	8.4

* Values in the table are the column percentage (%).

† Includes students, daily labourers and unemployed.

‡ Use of any substances, such as alcohol, khat or cigarettes.

§ Includes any of the following obstetric problems in previous pregnancy: abortion, stillbirth, preterm birth, delivery complications, CS delivery, prolonged labour, congenital anomalies and neonatal deaths.

¶ Includes any of the following pregnancy danger signs: severe or persistent headache, vaginal bleeding, fever, severe abdominal pain, difficulty breathing, convulsions or seizures, loss of consciousness, blurring of vision, and noticing the baby has completely stopped moving.

** Including any of the following in the current pregnancy: pre-eclampsia, hyperemesis gravidarum, oligo/polyhydramnios, RH isoimmunisation.

†† Including any of the following chronic illnesses, such as women with current diagnosis of syphilis, HIV, DM, HPN, at enrolment and self-reported history of DM, HPN, cardiac problem, mental health problem, HIV, hepatitis, renal diseases, and other issues like asthma, PID, gastroenteritis, haemorrhoid, ovarian cyst, PUD, STI, goitre and uterine myoma.

HgB, Haemoglobin; MUAC, mid-upper arm circumference.

### Longitudinal pattern of antenatal depressive symptoms and the receipt of care

Table 3 shows the prevalence of antenatal depressive symptoms at each measurement point. The results showed that 226 (24.2%) pregnant women at the first measurement time, 344 (36.9%) at the second, 276 (32.3%) at the third and 204 (28.0%) at the fourth experienced antenatal depressive symptoms. Overall, 30.5% (95% CI 29.0 to 31.8) of women experienced antenatal depressive symptoms (table 3).

**Table 3:** Longitudinal pattern of antenatal depressive symptoms, maternal and behavioural health indicators across measurement points among pregnant women (n=4279)

Survey time	Interviewed (n)	Depressive symptoms n (%)		Pregnancy danger signs n (%)*		Substance use n (%) †
		PHQ-9 ≥5	PHQ-9 ≥10	At least one	At least two	
Time 1	933	226 (24.2)	61 (6.5)	316 (33.9)	134 (14.4)	227 (24.3)
Time 2	933	344 (36.9)	120 (12.9)	478 (51.2)	234 (25.1)	166 (17.8)
Time 3	854	276 (32.3)	79 (9.3)	431 (50.5)	210 (24.6)	154 (18.0)
Time 4	728	204 (28.0)	67 (9.2)	349 (47.9)	178 (24.5)	130 (17.9)
Time 5	506	151 (29.8)	46 (9.1)	246 (48.5)	119 (23.5)	104 (20.6)
Time 6	240	75 (31.3)	22 (9.2)	108 (45.0)	51 (21.3)	39 (16.3)
Time 7	85	28 (32.9)	7 (8.2)	37 (43.0)	17 (20.0)	8 (9.4)
Total	4279	1330 (30.5)	402 (9.4)	1965 (45.9)	943 (22.0)	828 (19.4)

Values in the table are row percentages (%).

* Including any of the following pregnancy danger signs: severe or persistent headache, vaginal bleeding, fever, severe abdominal pain, difficulty breathing, convulsions or seizures, loss of consciousness, blurring of vision, and noticing the baby has completely stopped moving.

† Use of any substances, such as alcohol, khat or cigarettes, in the current pregnancy.

PHQ-9, Patient Health Questionnaire-9.

Figure 1 presents women’s antenatal depressive symptoms by measurement point and trimester level. The results showed that women’s antenatal depressive symptoms were higher in their first trimester, both at baseline (35.6%) and during the follow-up period (37.2%) (figure 1). Figure 2 presents the cumulative prevalence of antenatal depressive symptoms; more than half of the participants (59.1%) experienced antenatal depressive symptoms at least once, 349 (37.4%) twice, and 215 (23.1%) three or more times. Despite this burden, only 52 (5.6%) pregnant women received optimal care for depressive symptoms, including psychological counselling, therapy, or social support from healthcare providers, religious leaders, or community organisations (figure 2).

**Figure 1:**
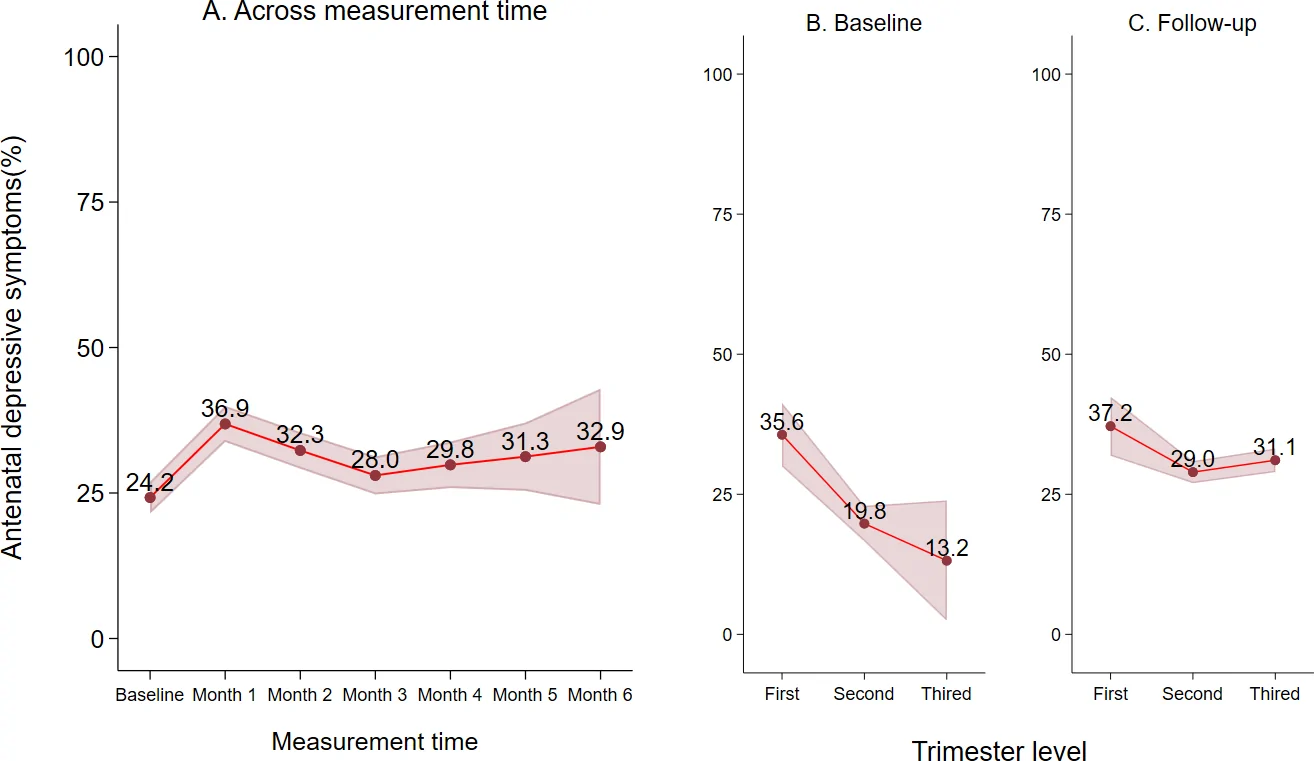
Women’s antenatal depressive symptoms by measurement point and their trimester level.

**Figure 2:**
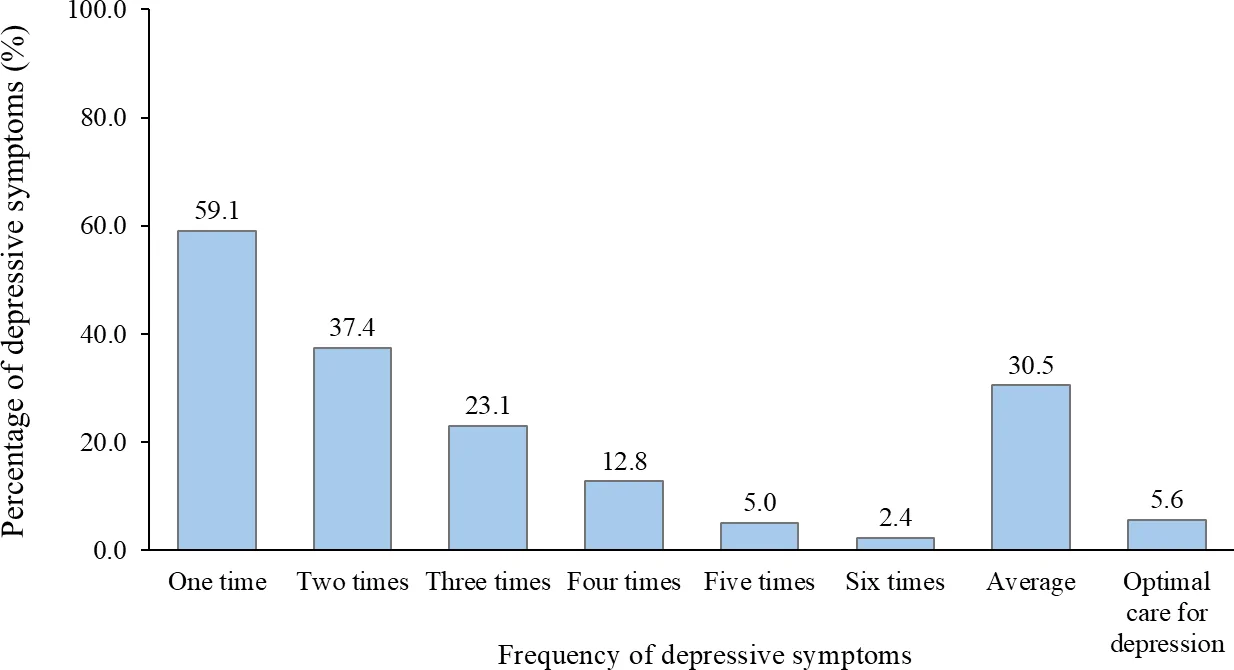
Cumulative percentage of antenatal depressive symptoms across seven measurement time points among pregnant women.

### Trajectory of antenatal depressive symptoms

Table 4 shows the fit statistics and class membership of the trajectory model. The results showed that the three-class model had lower BIC and AIC values and classified participants with proportions greater than 5%, with the average posterior probability (AvePP) of each class and the entropy values within acceptable ranges. Additionally, this model is clinically important for distinguishing trajectories between normal, mild, and moderate-to-high depressive symptoms, and the classes had narrower CIs. The two-class, four-class and five-class models demonstrated acceptable entropy and AvePP values and provided good classification of participants in each class except for the five-class model. However, the trajectory plots showed wide CIs and were less effective at distinguishing trajectories between mild and mild-to-moderate depressive symptoms over time, with some overlap (four-class and five-class models) and a wide confidence interval (two-class model) (table 4). Consequently, the three-class quadratic polynomial order model was selected to illustrate the trajectory of antenatal depressive symptoms over time.

**Table 4:** Fit statistics and class membership of the trajectory model results

Class	AIC	BIC	Entropy	AvePP range*	Minimum class proportion (%)
2	−9939.4	−9958.8	0.76	0.89–0.95	29.7
3	−9785.9	−9814.9	0.72	0.86–0.92	11.6
4	−9741.9	−9780.7	0.71	0.79–0.88	7.7
5	−9695	−9743.4	0.75	0.80–0.93	1.4

* Average posterior probability of each group.

AIC, Akaike Information Criterion; AvePP, average posterior probability; BIC, Bayesian Information Criterion.

Figure 3 presents the three-class trajectory model of antenatal depressive symptoms. The results showed that 356 (38.1%) of pregnant women who exhibited minimal depressive symptoms (Class 1) with the mean PHQ-9 score between 1 to 2, 469 (50.3%) of pregnant women exhibited mild with slight fluctuations in depressive symptoms (Class 2) with mean PHQ-9 score started from 4 at baseline and raised slightly to 5 at second time point, dropped back to 4 at time four and slightly increased to 4.5 at the sixth time point; and 108 (11.6%) of pregnant women exhibited mild-to-moderate steadily increased depressive symptoms (Class 3) with a mean baseline PHQ-9 score of 6.5, and increased steadily to 12 at time four and then decreased slightly to 10.5 at time six (figure 3).

**Figure 3:**
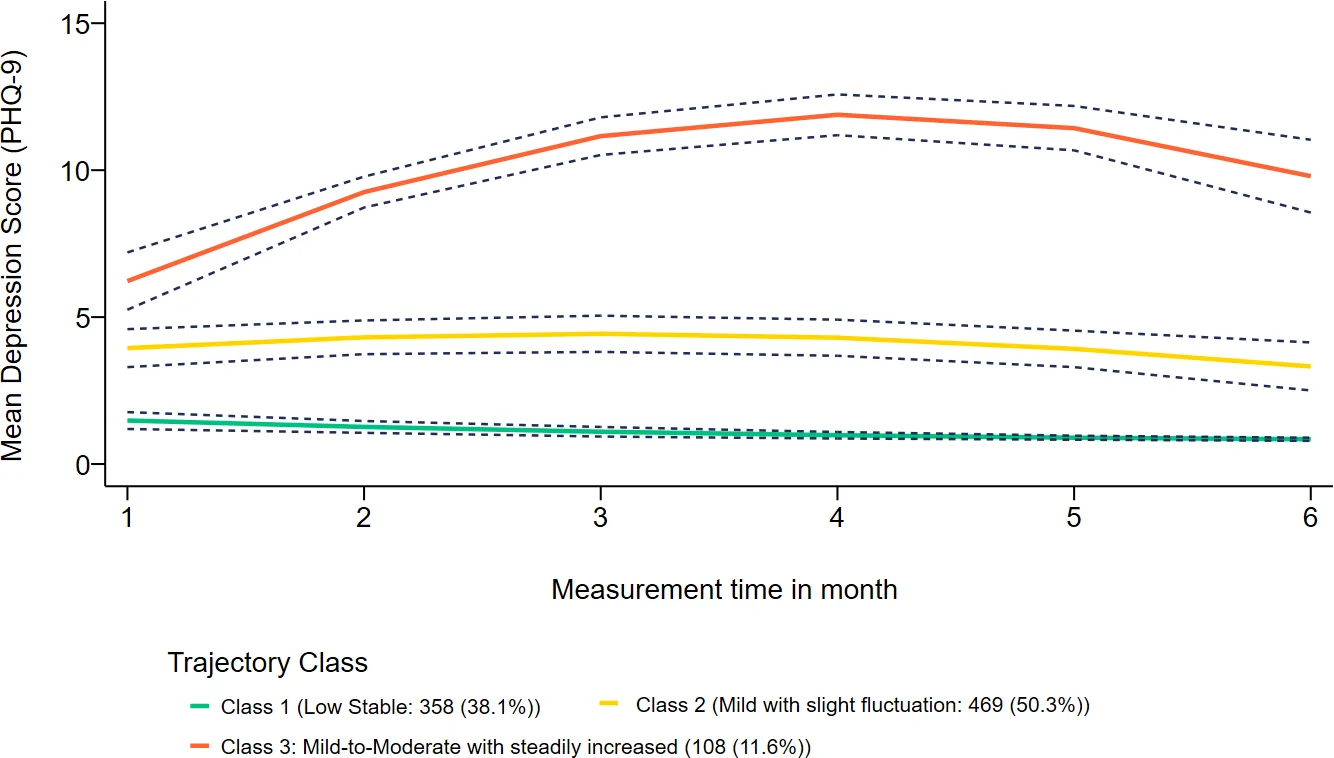
The estimated trajectories of antenatal depressive symptoms over time among pregnant women. PHQ-9, Patient Health Questionnaire.

### Factors associated with antenatal depressive symptoms

Before identifying the determinants, attrition bias was assessed, and the results showed a significant difference between completers and dropouts across several variables, including antenatal depressive symptoms, marital status, educational status, wealth index, pregnancy intention and women’s health-related quality of life (HRQoL) (all p values <0.05). Looking at standardised difference values, three variables, including antenatal depressive symptoms, wealth index and pregnancy intention, exceeded the threshold value (0.2) and significantly predicted dropout, which showed a meaningful imbalance (online supplemental table 2).

Furthermore, we conducted a competing risk analysis to determine whether the participants’ baseline characteristics predict study attrition, and the multivariable Fine-Gray subdistribution hazard model result revealed that women who experienced depressive symptoms at baseline, were currently married, belonged to rural residence, were formally employed, and reported high HRQoL significantly predicted a lower rate of lost follow-up or a higher rate of retention. In contrast, women who’s aged <20 years and between 20 and 34 years, belonging to poorest wealth index (adjusted Subdistribution Hazard Ratio (aSHR)=2.03; 95% CI 1.53 to 2.68), women enrolled at first trimester (aSHR=2.82; 95% CI 1.51 to 5.25), those reported their health status as moderate/poor (aSHR=1.61; 95% CI 1.32 to 1.98) and those who experienced at least one obstetric complications (aSHR=1.49; 95% CI 1.19 to 1.87) were significantly increased the rate lost follow-up or study attrition (online supplemental table 3).

Table 5 shows the determinants of antenatal depressive symptoms. After adjusting for confounders, currently married pregnant women were less likely to experience antenatal depressive symptoms (aOR=0.53; 95% CI 0.28 to 0.98). The odds of antenatal depressive symptoms were higher among those women who had an unintended pregnancy (aOR=1.29; 95% CI 1.03 to 1.62), experienced intimate partner violence (aOR=2.43; 95% CI 1.57 to 3.75) and those who experienced pregnancy danger signs (aOR=2.76; 95% CI 2.28 to 3.34). Furthermore, pregnant women at first trimester had 1.53 times higher odds of experiencing antenatal depressive symptoms than those in their third trimester (aOR=1.53; 95% CI 1.11 to 2.13), and pregnant women with moderate/low health-related quality of life had significantly higher odds of antenatal depressive symptoms than those with high health-related quality of life (aOR=2.43; 95% CI 1.78 to 3.32) (table 5).

**Table 5:** Determinants of antenatal depressive symptoms among pregnant women, results of generalised estimating equations (n=4044 observations)

Variables	Depressive symptoms*		cOR, 95% CI	aOR, 95% CI
	Experienced (%)	Not experienced (%)		
Educational status				
No education or some primary	33.6	66.4	1.24 (0.95 to 1.62)†	0.99 (0.74 to 1.34)
Complete primary or some secondary	28.4	71.6	0.99 (0.76 to 1.29)	0.81 (0.62 to 1.06)
Complete secondary or higher	28.7	71.3	1	1
Marital status				
Currently married	30.0	69.0	0.45 (0.23 to 0.87)‡	0.53 (0.28 to 0.98)‡
Currently not married	52.1	47.9	1	1
Wealth index				
Poorest	33.0	66.0	1.29 (1.01 to 1.64)‡	1.13 (0.86 to 1.48)
Middle	30.4	69.6	1.13 (0.88 to 1.44)	1.01 (0.79 to 1.29)
Richest	28.2	71.8	1	1
Religion				
Christian	32.2	67.8	1.71 (0.99 to 1.96)†	1.31 (0.71 to 2.41)
Muslim	26.4	73.6	1.25 (0.70 to 2.22)	0.93 (0.49 to 1.74)
Others§	22.6	77.4	1	1
Trimester level				
First trimester	37.0	63.0	1.25 (0.96 to 1.63)†	1.45 (1.09 to 1.93)‡
Second trimester	29.0	71.0	0.91 (0.81 to 1.03)†	0.92 (0.80 to 1.06)
Third trimester	31.0	69.0	1	1
Pregnancy intention				
Unintended	35.4	64.6	1.36 (1.09 to 1.68)¶	1.29 (1.03 to 1.62)‡
Intended	28.8	71.2	1	1
Experience of intimate partner violence				
Yes	56.2	43.8	3.09 (2.11 to 4.53)**	2.43 (1.67 to 3.56)**
No	28.7	72.3	1	1
Self-reported health status				
Poor, fair, good	35.9	64.1	1.88 (1.61 to 2.19)**	1.66 (1.40 to 1.97)**
Very good, excellent	20.9	79.1	1	1
Health-related quality of life				
Moderate/low	53.5	46.5	3.08 (2.25 to 4.23)**	2.43 (1.78 to 3.32)**
High	28.3	71.7	1	1
Experience of pregnancy danger signs††				
Yes	46.3	53.7	2.83 (2.44 to 3.28)**	2.60 (2.22 to 3.04)**
No	16.9	83.1	1	1
Experience of pregnancy complications‡‡				
Yes	49.8	50.2	2.06 (1.71 to 2.48)**	1.87 (1.53 to 2.29)**
No	27.2	72.8	1	1

* Values in the table are row percentages.

† p<0.2.

‡ p<0.05.

§ Includes Adventist and traditional.

¶ p<0.01.

** p<0.001.

†† Includes severe or persistent headache, vaginal bleeding, fever, severe abdominal pain, difficulty breathing, convulsions or seizures, loss of consciousness, blurring of vision, and noticing the baby completely stopped moving.

‡‡ Includes any of preeclampsia, hyperemesis gravidarum, oligo/polyhydramnios, and Rh isoimmunization.

1, reference group; aOR, adjusted odds ratio; cOR, Crude odds ratio.

The sensitivity analysis results comparing the full-sample model with participants who completed all six time points revealed that most predictors maintained consistent direction. At the same time, the wealth index variable showed a reverse direction and a 38% change in the OR. The four variables, such as marital status, religion, intimate partner violence (IPV) and trimester level, exhibited more than 30% OR changes between the two models (online supplemental table 4). These findings suggest that attrition may substantially bias the GEE results and that the results should be interpreted with caution in later pregnancy.

A sensitivity analysis restricted to those who enrolled in their first trimester and were followed until the end of pregnancy (up to six time points) (Model II), and those who completed all four rounds regardless of their trimester level (Model III), showed that there is no directional change in association compared with the original model or full sample model (Model I). However, there was a significant effect size change in marital status between the final full sample model (Model I) and those who completed only four rounds (Model III), with an aOR change >30% (online supplemental table 5). Furthermore, another sensitivity analysis comparing participants with PHQ-9 ≥10 as defining the experience of depressive symptoms showed that only two variables, including pregnancy intention and women’s gestation age, did not show a significant association with antenatal depressive symptoms, while the relationships of the remaining variables were similar to the model with a PHQ-9 ≥5 (online supplemental table 6).

## Discussion

In this study, close to one-third of pregnant women experienced antenatal depressive symptoms. One in 10 pregnant women had a trajectory of mild-to-moderate with steadily increasing depressive symptoms, and half of the participants had a mild trajectory with slightly fluctuating depressive symptoms (50.3%). Both pregnancy-related and psychosocial factors were positively associated with the experience of antenatal depressive symptoms. The interpretation of women’s depressive symptoms was based on a PHQ-9 score≥5, focused on the broad spectrum of depressive symptoms screening, enabling the detection of mild-to-moderate depressive symptoms and subclinical depressive symptoms rather than a clinical diagnosis of depression (PHQ-9 ≥10), which was validated among pregnant women,^54^ and in primary care settings in Ethiopia.^55^ Consequently, this study’s findings reflect the overall burden of depressive symptoms rather than clinical depression.

The present study found that 30.5% of pregnant women experienced antenatal depressive symptoms. This high burden of antenatal depressive symptoms is comparable to other studies conducted in the Maichew^56^ and Sodo Districts of Ethiopia.^7 31^ Antenatal depressive symptoms were exacerbated by the COVID-19 pandemic, which imposed several psychosocial distress^57^ alongside substantial social and economic impacts,^58^ which led to increased antenatal depressive symptoms.^59 60^ Although we did not collect individual COVID-19 data in this study, our study findings align with both pre-pandemic^61–63^ and post-pandemic studies^64 65^ conducted in similar settings.

However, this study’s findings are higher than those of studies conducted in Jimma Town and Gida Ayana District,^31 32^ Woliso District,^66^ Gondar Town,^21^ Debre Tabor Town,^27 67^ Dessie Town^68^ and Fafen Zone,^29^ ranging from 6.9 in Gondar Town to 24.4% in the Fafen Zone. This variation could be attributed to methodological differences, as some studies measured depressive symptoms at once during pregnancy and used varied measurement tools with different cut-off points.^27 56 66–68^ Additionally, studies conducted in Gondar and Dessie Town primarily focused on women in their second and third trimesters,^21 68^ while other studies^21 29 31 32 66–68^ included less than 10% of participants in their first trimester. Women in their first trimester encounter pregnancy-related problems, such as nausea, vomiting and hyperemesis, leading to increased depressive symptoms.^69^

Furthermore, the current finding is comparable to other countries’ findings conducted in Ghana,^70^ Rwanda^70^ and India,^69^ but lower than studies conducted in Ghana,^71^ Uganda,^72 73^ Kenya^74^ and India,^75^ ranging from 36% to 41%. This variation could be attributed to variations in measurement tools^75^ and differences among participants,^71 73 74^ conducted on adolescent pregnant women, who are highly vulnerable to depressive symptoms and a high burden of pregnancy-related complications such as pre-eclampsia, anaemia and fatigue.^76^ These health problems increase the risk of experiencing depressive symptoms.

Despite the regional and international similarities and variations, this study measured depressive symptoms at multiple time points throughout the pregnancy, providing a realistic estimate of antenatal depressive symptom burden. However, generalisability may be limited because participants were recruited solely from healthcare facilities, and depressive symptoms were self-reported, which may have led to misclassification of pregnancy-related issues as depressive symptoms. Furthermore, given the sensitive nature of maternal mental health, using self-reported measures and collecting follow-up data through cell phones may have introduced a social desirability bias, as well as overestimating or underestimating the burden of depressive symptoms.

Depressive symptoms varied over the period of pregnancy, and we assessed the distinct pattern of depressive symptoms across the six measurement points. Consequently, this study revealed that 11.6% of pregnant women demonstrated a mild-to-moderate level with steadily increasing depressive symptoms over time, which is consistent with studies conducted in the Netherlands^77^ and in Brazil.^78^ However, it is higher than a facility-based cohort study conducted in Kenya^79^ and Canada.^80^ This difference could be attributed to variations in trajectory class, measurement tool and participant characteristics. Consequently, our findings have implications for women’s health by hindering their health-promoting behaviours, including poor stress management, inadequate nutrition intake, decreased healthcare utilisation and poor adherence to healthcare advice,^12 81^ as well as problems with feto-maternal attachments,^82^ which potentially cause various adverse perinatal outcomes such as abortion, labour complications, stillbirth and preterm births.^7 83^

The present study also identified different sociodemographic, pregnancy, clinical and psychosocial-related factors affecting antenatal depressive symptoms, and the findings showed that the odds of experiencing antenatal depressive symptoms were reduced by 53% among currently married participants, which is consistent with other cross-sectional studies conducted in Ethiopia,^28 56^ Ghana^71^ and Nigeria.^84^ This association could be due to a healthy marriage that has good support from the spouse and reduces the likelihood of financial and social instabilities, which ultimately reduces the experience of depressive symptoms.^85^

IPV is the most common psychological factor that many women face during pregnancy, and the positive relationship between IPV and depressive symptoms has been clearly documented in several studies in Ethiopia,^30 68 86 87^ Tanzania,^88–90^ Malawi,^91^ Uganda,^72^ India,^75^ Bangladesh and^92 93^ Nepal,^94^ which are consistent with our current study findings, revealed that pregnant women who experienced intimate partner violence were positively associated with the experience of antenatal depressive symptoms. This association could be attributed to the fact that IPV contributes to the breakup of relationships, social isolation, self-stigmatisation and poor or unhealthy lifestyles,^95^ which leads to experiencing depressive symptoms.

Women who had an unintended pregnancy had 29% higher odds of experiencing antenatal depressive symptoms as compared with their counterparts. This finding was similar to other facility-based and community-based studies conducted in different districts of Ethiopia, such as Debre Tabor,^27 67^ Aneded^28^ and Gida Ayana,^32^ which showed that the experience of antenatal depressive symptoms increased by more than threefold among those who had an unintended pregnancy. Similarly, other facility-based and community-based studies in Uganda,^72^ Nepal^94^ and Bangladesh^92^ have shown that unintended pregnancy has been strongly associated with the experiences of antenatal depressive symptoms. This similarity across findings could be attributed to the presence of shared psychological and structural factors among women with unintended pregnancy, such as exposure to stress, financial difficulties, inadequate support from partners and the presence of marital conflict^96^ which increases the risk of developing antenatal depressive symptoms.^32 67 73 97^

The present study findings showed that first-trimester women had higher odds of experiencing antenatal depressive symptoms than third-trimester women, which is in line with a cross-sectional study conducted in Ethiopia and Uganda.^72 98 99^ This could be attributed to women in the first trimester being troubled with different pregnancy-related problems, mainly nausea, vomiting and hyperemesis gravidarum,^100^ which increases depressive symptoms. Women who experienced pregnancy complications had 87% higher odds of experiencing antenatal depressive symptoms, which is consistent with a community-based and facility-based study conducted in Ethiopia^67^ and Gahan.^70^ This association could be attributed to the presence of fear or stress from experiencing pregnancy-related problems that may lead to birth-related complications.^27 101^

Furthermore, this study’s findings showed that women who experienced any of the pregnancy danger signs were nearly threefold more likely to experience antenatal depressive symptoms. This relationship has not been documented in previous studies; however, a community-based cohort study conducted in Ethiopia reported that antenatal depressive symptoms were positively associated with the experience of perinatal complications, including pregnancy red flags and labour complications.^7^ Consequently, the current and previous studies highlighted that pregnancy danger signs and antenatal depressive symptoms had a bidirectional relationship.

Moreover, the present study findings revealed that women who reported low-to-moderate health-related quality of life were positively associated with the experience of antenatal depressive symptoms, which is supported by case-control and cross-sectional studies conducted in Iran and China.^102 103^ Additionally, findings from the current study showed that women who rated their current health status as good or lower had 66% higher odds of experiencing antenatal depressive symptoms as compared with those who reported it as very good or excellent. The finding was supported by a cross-sectional facility-based study conducted in Rwanda.^97^ This positive association could be that women rated as having poor/fair health status may reflect experiencing pregnancy-related problems, lack of access to healthcare, lack of support from the family and spouse, and nutritional issues, as well as socioeconomic disparities. Consequently, the cumulative burden of these problems may lead women to experience antenatal depressive symptoms.

## Conclusions

In this study, antenatal depressive symptoms were common and more prevalent; nearly one in three pregnant women experienced antenatal depressive symptoms, while more than one in 10 (11.6%) pregnant women had steadily increasing mild-to-moderate depressive symptoms over time. Factors included unintended pregnancy, experiencing intimate partner violence, pregnancy danger signs, pregnancy complications, being in the first trimester, self-rated poor health and moderate/low health-related quality of life were identified as the main determinants of antenatal depressive symptoms. The findings underscore the importance of ongoing screening for depressive symptoms using locally validated tools during pregnancy, and the integration of treatment cascades into women’s continuum of care. Psychosocial and pregnancy-related screening, along with appropriate psychosocial support, should be strengthened during ANC visits. Strategic and policy interventions, including psychosocial support, interpersonal therapy and a healthy lifestyle for addressing depressive symptoms, partner violence and behavioural health issues, should be enhanced.

## Supplementary material

10.1136/bmjopen-2026-120181 online supplemental table 1

10.1136/bmjopen-2026-120181 online supplemental table 2

10.1136/bmjopen-2026-120181 online supplemental table 3

10.1136/bmjopen-2026-120181 online supplemental table 4

10.1136/bmjopen-2026-120181 online supplemental table 5

10.1136/bmjopen-2026-120181 online supplemental table 6

## Data Availability

Data are available in a public, open access repository.
