# Supplementary material for: Magnitude, trajectory and determinants of antenatal depressive symptoms in Adama Town and East Shewa Zone, Ethiopia: evidence from a prospective longitudinal eCohort survey
Source: BMJ Open. 2026 Oct 7;16(10):e120181. doi: 10.1136/bmjopen-2026-120181 (PMC13646975; doi:10.1136/bmjopen-2026-120181)
Supplement: Supplementary file 1 [file bmjopen-16-10-s001.pdf]

Supplementary Table 1: The estimated within-subject correlation matrix of antenatal depressive symptoms and model comparison of working correlation structure

	**c1**	**c2**	**c3**	**c4**	**c5**	**c6**
r1	1					
r2	0.238	1				
r3	0.194	0.492	1			
r4	0.186	0.371	0.54	1		
r5	0.122	0.353	0.427	0.475	1	
r6	0.149	0.357	0.469	0.553	0.544	1

Working correlation	Metric indices value (QIC)
Exchangeable	4088
Autoregressive (AR (1))	4092
Unstructured	4103
