# Supplementary material for: Magnitude, trajectory and determinants of antenatal depressive symptoms in Adama Town and East Shewa Zone, Ethiopia: evidence from a prospective longitudinal eCohort survey
Source: BMJ Open. 2026 Oct 7;16(10):e120181. doi: 10.1136/bmjopen-2026-120181 (PMC13646975; doi:10.1136/bmjopen-2026-120181)
Supplement: Supplementary file 2 [file bmjopen-16-10-s002.pdf]

Supplementary Table 2: Participants' baseline characteristics by completion status and multivariable logistic regression predicting dropout

Baseline variables	Dropout [b] Yes, n (%)	Dropout [b] No, n (%)	Chi2	Std Diff	aOR, 95%CI	P-value
Depressive symptoms						
Experienced	148 (65.5)	78 (34.5)	0.000	0.3267	0.53 (0.36-0.79)	0.002
Not experienced	564 (79.8)	143 (20.2)			1	
Age						
< 20 Years	67 (84.8)	12 (15.2)	0.061	0.0447	1.21 (0.43-3.35)	0.719
20-34 years	559 (75.0)	200 (25.0)			0.82 (0.38-1.79)	0.628
>30 Years	46 (83.6)	9 (16.4)			1	
Marital status						
Currently married	691 (75.9)	220 (24.2)	0.033	0.1938	0.18 (0.02-1.43)	0.105
Not currently married	21 (95.5)	1 (4.6)			1	
Residence						
Urban	338 (73.8)	120 (26.2)	0.076	-0.1367	1.28 (0.85-1.94)	0.233
Rural	374 (78.7)	101 (21.3)			1	
Educational status						
No education or some primary	289 (81.2	67 (18.8)			1.01 (0.60-1.70)	0.956
Complete primary or some secondary	275 (73.7)	98 (26.3)	0.022	0.1971	0.89 (0.57-1.39)	0.605
Complete secondary or higher	148 (72.6)	56 (27.5)			1	
Wealth index						
Poorest	256 (84.2)	48 (15.8)			2.10 (1.22-3.60)	0.007
Middle	224 (73.7)	80 (26.3)	0.000	0.2981	1.24 (0.81-1.89)	0.322
Richest	229 (76.4)	88 (28.7)			1	
Religion						
Christian	500 (75.9)	159 (24.1)			1.41 (0.56-3.53)	0.466
Muslim	189 (77.5)	55 (22.5)	0.882	-0.0336	1.26 (0.49-3.24)	0.626
Others	23 (76.7)	7 (23.3)			1	
Occupation						
Employed	126 (71.6)	50 (28.4)			0.67 (0.30-1.47)	0.317
Merchant	104 (74.3)	36 (25.7)	0.185	-0.1637	0.77 (0.35-1.71)	0.526
Housewife	422 (77.4)	123 (22.6)			0.82 (0.41-1.68)	0.605
Others	60 (83.3)	12 (16.7)			1	
Pregnancy intention						
Unintended	212 (85.1)	37 (14.9)	0.000	0.3127	1.85 (1.21-2.83)	0.004
Intended	499 (73.1)	184 (26.9)			1	
Experience of intimate partner violence						
Yes	47 (79.7)	12 (20.3)	0.532	-0.0492	1.31 (0.64-2.66)	0.462
No	665 (76.1)	209 (23.9)			1	
Self-reported health status						
Poor/fair/good	435 (74.1)	152 (25.9)	0.039	-0.1613	1	0.211
Very good/excellent	277 (80.06)	69 (19.9)			1.25 (0.88-1.80)	
Malnutrition						
Yes	154 (76.2)	48 (23.8)	0.993	0.0007	0.99 (0.66-1.49)	0.96
No	556 (76.3)	173 (23.7)			1	
Anemia						
Yes	75 (79.8)	19 (20.2)	0.412	-0.0665	1.33 (0.75-2.37)	0.33
No	598 (76.0)	189 (24.0)			1	
Women HRQoL						
Moderate/low	51 (65.4)	27 (34.6)	0.018	-0.1712	0.86 (0.48-1.53)	0.599
High	661 (77.3)	194 (22.7)			1	
Experience of pregnancy danger signs [b]						
At least one	231 (78.3)	64 (21.7)	0.325	0.1717	0.77 (0.52-1.15)	0.2
No	477 (75.4)	156 (24.6)			1	
Experience of obstetric problems [c]						
Yes	100 (70.9)	41 (29.1)	0.102	0.1221	0.90 (0.58-1.40)	0.632
No	612 (77.3)	180 (22.7)			1	

[a] Value in the table is row percentage, [b] Including severe or persistent headache, vaginal bleeding, fever, severe abdominal pain, difficulty breathing, convulsion or seizure, loss of consciousness, blurring of vision, and noticing the baby completely stopped moving. [c] Including any of preeclampsia, hyperemesis gravidarum, oligo/polyhydramnios, and RH-isoimmunization.
