# Supplementary material for: Magnitude, trajectory and determinants of antenatal depressive symptoms in Adama Town and East Shewa Zone, Ethiopia: evidence from a prospective longitudinal eCohort survey
Source: BMJ Open. 2026 Oct 7;16(10):e120181. doi: 10.1136/bmjopen-2026-120181 (PMC13646975; doi:10.1136/bmjopen-2026-120181)
Supplement: Supplementary file 3 [file bmjopen-16-10-s003.pdf]

Supplementary Table 3: Predictors of true lost follow-up accounting for lost because of delivery/abortion/stillbirth as a competing event among pregnant women

Baseline variables	Model I		Model II	
	**Unadjusted sHR, 95% CI**	**Adjusted sHR, 95% CI**	**Unadjusted sHR, 95% CI**	**Adjusted sHR, 95% CI**
Depressive symptoms				
Experienced	0.95 (0.73-1.22)	0.70 (0.52-0.96) [b]	1.09 (0.90-1.32)	0.67 (0.54-0.84) [c]
Not experienced	1	1	1	1
Age				
< 20 Years	0.96 (0.52-1.75)	1.21 (0.75-1.97)	1.88 (1.14-3.10) [b]	1.71 (1.02-2.87) [b]
20-34 years	1.07 (0.67-1.71)	0.86 (0.44-1.68)	1.62 (1.05-2.50)	1.78 (1.15-2.76) [c]
≥ 35 years	1	1	1	1
Marital status				
Currently married	0.50 (0.28-0.91) [b]	0.37 (0.19-0.71) [c]	0.61 (0.40-0.94) [b]	0.56 (0.35-0.92) [b]
Not currently married	1	1	1	1
Residence				
Urban	1	1	1	1
Rural	0.79 (0.64-0.99) [b]	0.63 (0.48-0.82) [c]	0.92 (0.78-1.09)	0.69 (0.56-0.84) [d]
Educational status				
No education or some primary	0.80 (0.61-1.04)	0.62 (0.46-0.83) [c]	1.24 (0.99-1.56) [a]	1.09 (0.83-1.43)
Completed primary	0.58 (0.44-0.77) [d]	0.41 (0.30-0.55) [d]	1.11 (0.88-1.40)	0.90 (0.70-1.16)
Completed secondary or higher	1	1	1	1
Wealth index				
Poorest	0.91 (0.70-1.17)	1.30 (0.91-1.87)	1.28 (1.05-1.57) [b]	2.03 (1.54-2.49) [d]
Middle	0.68 (0.52-0.89) [c]	0.96 (0.71-1.26)	1.13 (0.91-1.39)	1.57 (1.25-1.97)
Richest	1	1	1	1
Religion				
Christian	1	1	1	1
Muslim	1.20 (0.95-1.52)	1.09 (0.84-1.43)	0.87 (0.71-1.05) [a]	0.78 (0.63-0.98) [b]
Others [e]	0.19 (0.05-0.77) [b]	0.22 (0.06-0.90) [b]	0.36 (0.17-0.77) [c]	0.36 (0.17-0.73) [c]
Occupation				
Employed	0.42 (0.25-0.70) [c]	0.33 (0.19-0.56) [d]	0.55 (0.39-0.76) [d]	0.49 (0.34-0.70) [d]
Merchant	0.85 (0.54-1.34)	0.76 (0.47-1.22)	0.81 (0.59-1.11)	0.77 (0.56-1.06)
Housewife	0.92 (0.63-1.36)	0.86 (0.57-1.29)	0.65 (0.49-0.85) [c]	0.54 (0.40-0.71)
Others [f]	1	1	1	1
Gestational age				
First trimester	1.94 (0.79-4.75) [a]	2.58 (0.93-7.17)	2.41 (1.41-4.13) [c]	2.82 (1.51-5.25) [c]
Second trimester	1.32 (0.54-3.22)	1.51 (0.56-4.04)	1.47 (0.86-2.50) [a]	1.46 (0.81-2.64)
Third trimester	1	1	1	
Pregnancy intention				
Unintended	1.58 (1.26-1.99) [d]	1.66 (1.27-2.17) [d]	0.99 (0.83-1.21)	1.06 (0.86-1.31)
Intended	1	1	1	1
Experience of intimate partner violence				
Yes	0.68 (0.40-1.17) [a]	0.60 (0.35-1.04)	0.99 (0.70-1.39)	0.88 (0.63-1.24)
No	1	1	1	1
Self-reported health status				
Poor/fair/good	1.21 (0.96-1.53) [a]	1.35 (1.04-1.75) [b]	1.38 (1.15-1.65) [d]	1.61 (1.32-1.98) [d]
Very good/excellent	1	1	1	1
Malnutrition (MUAC< 23 CM)				
Yes	0.72 (0.54-0.97) [b]	0.73 (0.53-1.01)	0.90 (0.73-1.11)	0.87 (0.69-1.08)
No	1	1	1	1
Anemia (Hbl <11 G/dL)				
Yes	1.19 (0.84-1.68)	1.61 (1.08-2.41) [b]	0.85 (0.63-1.14)	1.11 (0.80-1.52)
No	1	1	1	1
Women HRQoL				
Moderate/low	1	1	1	1
High	0.69 (0.49-0.97) [b]	0.61 (0.42-0.89) [b]	0.68 (0.52-0.88) [c]	0.59 (0.44-0.80) [c]
Experience of pregnancy danger signs [g]				
At least one	1.11 (0.88-1.39)	1.08 (0.85-1.38)	1.22 (1.03-1.44) b	0.92 (0.77-1.11)
No	1	1	1	1

Experience of obstetric problems [h]				
Yes	1.59 (1.22-2.06) [c]	1.44 (1.07-1.94) [b]	1.61 (1.32-1.97) [d]	1.49 (1.18-1.87) [c]
No	1	1	1	1

Model I: Six-round follow-up. **Model II**: Seven-round follow-up. [1] Reference**.** [a] P-value < 0.25, [b] < 0.05, [c] < 0.01, [d] < 0.001, [e] Includes Adventist and traditional. [f] Includes student and daily labour. [g] Includes severe or persistent headache, vaginal bleeding, fever, severe abdominal pain, difficulty breathing, convulsion or seizure, loss of consciousness, blurring of vision, and noticing the baby completely stopped moving. [h] Including any of preeclampsia, hyperemesis gravidarum, oligo/polyhydramnios, and RH-isoimmunization.
