# Supplementary material for: Magnitude, trajectory and determinants of antenatal depressive symptoms in Adama Town and East Shewa Zone, Ethiopia: evidence from a prospective longitudinal eCohort survey
Source: BMJ Open. 2026 Oct 7;16(10):e120181. doi: 10.1136/bmjopen-2026-120181 (PMC13646975; doi:10.1136/bmjopen-2026-120181)
Supplement: Supplementary file 4 [file bmjopen-16-10-s004.pdf]

Supplementary Table 4: Sensitivity analysis comparing the full sample and six-time point completer GEE models for determinants of antenatal depressive symptoms

Variables	Model I		Model II	OR Change (%)
	**cOR, 95%CI**	**aOR, 95%CI**	**aOR, 95%CI**	
Educational status				
No education or some primary	1.24 (0.95-1.62) [a]	0.99 (0.74-1.34)	0.96 (0.57-1.62)	-3.0
Complete primary or some secondary	0.99 (.76-1.29)	0.81 (0.62-1.06)	0.60 (0.37-0.96) [b]	-25.9
Complete secondary or higher	1	1	1	
Marital status				
Currently married	0.45 (0.23-0.87) [b]	0.53 (0.28-0.98) [b]	0.08 (0.05-0.12) [d]	-84.9
Currently not married	1	1	1	
Wealth index				
Poorest	1.29 (1.01-1.64) [b]	1.13 (0.87-1.48)	0.70 (0.41-1.18)	-38.1
Middle	1.13 (.88-1.44)	1.01 (0.79 - 1.29)	0.96 (0.63-1.46)	-4.9
Richest	1	1	1	
Religion				
Christian	1.71 (0.99-1.96) [a]	1.31 (0.71-2.41)	1.72 (0.47-6.29)	31.3
Muslim	1.25 (0.70-2.22)	0.93 (0.49-1.74)	1.0 (0.26-3.81)	7.5
Others	1	1	1	
Gestational age				
First trimester	1.25 (0.96-1.63) [a]	1.45 (1.10-1.93) [c]	1.99 (1.31-3.03) [c]	37.2
Second trimester	0.91 (0.81-1.03) [a]	0.93 (0.80-1.06)	1.15 (0.89-1.49)	23.7
Third trimester	1	1	1	
Pregnancy intention				
Unintended	1.36 (1.09-1.68) [c]	1.29 (1.03-1.62) [b]	1.50 (0.90-2.48)	16.3
Intended	1	1	1	
Experience of intimate partner violence				
Yes	3.09 (2.11-4.53) [d]	2.43 (1.67-3.56) [d]	4.67 (1.78-12.23) [c]	92.2
No	1	1	1	
Self-reported health status				
Poor, fair, good	1.88 (1.61-2.19) [d]	1.66 (1.40-1.97) [d]	1.45 (1.06-1.98) [b]	-12.7
Very good, excellent	1	1	1	
Health-related quality of life				
Moderate/low < 0.85	3.08 (2.25-4.23) [d]	2.43 (1.78-3.32) [d]	1.84 (1.13-3.00) [b]	-24.3
High ≥ 0.85	1	1	1	
Experience of pregnancy danger signs [e]				
At least one	2.83 (2.44-3.28) [d]	2.60 (2.22-3.04 [d]	2.30 (1.76-3.01) [d]	-11.5
No	1	1	1	
Experience of obstetric problems [f]				
At least one	2.06 (1.71-2.48) [d]	1.87 (1.53-2.29) [d]	2.17 (1.55-3.05) [d]	16.0
No	1	1	1	
**Observations**	**4044**		**1284**	

Model I: the full-sample model, including the six time-point assessments. **Model II:** the completer model (those participants completed all six measurement points), 1: Reference group. [a] P-value < 0.25, [b] < 0.05, [c] < 0.01, [d] < 0.001. [e] Including severe or persistent headache, vaginal bleeding, fever, severe abdominal pain, difficulty breathing, convulsion or seizure, loss of consciousness, blurring of vision, and noticing the baby completely stopped moving. [f] Including any of preeclampsia, hyperemesis gravidarum, oligo/polyhydramnios, and RH-isoimmunization.
