# Supplementary material for: Magnitude, trajectory and determinants of antenatal depressive symptoms in Adama Town and East Shewa Zone, Ethiopia: evidence from a prospective longitudinal eCohort survey
Source: BMJ Open. 2026 Oct 7;16(10):e120181. doi: 10.1136/bmjopen-2026-120181 (PMC13646975; doi:10.1136/bmjopen-2026-120181)
Supplement: Supplementary file 5 [file bmjopen-16-10-s005.pdf]

**Supplementary Table 5**: Sensitivity analysis for determinants of antenatal depressive symptoms among pregnant women, results of Generalized Estimation Equation with exchangeable correlation structure.

| | Model I | | Model II | | Model III | |
|---|---|---|---|---|---|---|
| **Variable** | **cOR, 95%CI** | **aOR, 95%CI** | **cOR, 95%CI** | **aOR, 95%CI** | **cOR, 95%CI** | **aOR, 95%CI** |
| Educational status (Ref = Completed secondary or higher) | | | | | | |
| No education or some primary | 1.24 (0.95–1.62) [a] | 0.99 (0.74–1.34) | 1.91 (1.21–2.99) [c] | 1.48 (0.89–2.46) | 1.39 (1.03–1.88) [b] | 1.07 (0.77–1.48) |
| Complete primary or some secondary | 0.99 (0.76–1.29) | 0.81 (0.62–1.06) | 1.11 (0.73–1.70) | 0.95 (0.61–1.48) | 1.07 (0.79–1.45) | 0.89 (0.66–1.21) |
| Marital status (Ref = Currently not married) | | | | | | |
| Currently married | 0.45 (0.23–0.87) [b] | 0.53 (0.28–0.98) [b] | 0.11 (0.09–0.14) [d] | 0.10 (0.05–0.20) [d] | 0.27 (0.11–0.67) [c] | 0.38 (0.17–0.82) [b] |
| Wealth index (Ref = Richest) | | | | | | |
| Poorest | 1.29 (1.01–1.64) [b] | 1.13 (0.86–1.48) | 1.29 (0.85–1.97) [a] | 1.05 (0.65–1.68) | 1.34 (1.02–1.76) [b] | 1.17 (0.86–1.59) |
| Middle | 1.13 (.88–1.44) | 1.01 (0.79–1.29) | 1.05 (0.69–1.59) | 1.02 (0.68–1.54) | 1.16 (0.88–1.52) | 1.0 (0.76–1.33) |
| Religion (Ref = Others) | | | | | | |
| Christian | 1.71 (0.99–1.96) [a] | 1.31 (0.71–2.41) | | | | |
| Muslim | 1.25 (0.70–2.22) | 0.93 (0.49–1.74) | | | | |
| Gestational age (Ref = Third trimester) | | | | | | |
| First trimester | 1.25 (0.96–1.63) [a] | 1.45 (1.09–1.93) [b] | 1.40 (1.02–1.93) [b] | 1.61 (1.12–2.30) [c] | 1.31 (0.98–1.75) [a] | 1.63 (1.19–2.23) [c] |
| Second trimester | 0.91 (0.81–1.03) [a] | 0.92 (0.80–1.06) | 1.18 (0.94–1.48) | 1.17 (0.90–1.52) | 0.93 (0.79–1.09) | 0.98 (0.82–1.18) |
| Pregnancy intention (Ref = Intended) | | | | | | |
| Unintended | 1.36 (1.09–1.68) [c] | 1.29 (1.03–1.62) [b] | 1.45 (0.96–2.19) [a] | 1.79 (1.11–2.88) [b] | 1.46 (1.13–1.89) [c] | 1.38 (1.05–1.81) [b] |
| Experience of intimate partner violence (Ref = No) | | | | | | |
| Yes | 3.09 (2.11–4.53) [d] | 2.43 (1.67–3.56) [d] | 4.15 (1.54–11.15) [c] | 4.27 (1.57–11.59) [c] | 3.08 (1.99–4.76) [d] | 2.51 (1.63–3.85) [d] |
| Self-reported health status (Ref = Very good/excellent) | | | | | | |
| Poor, fair, good | 1.88 (1.61–2.19) [d] | 1.66 (1.40–1.97) [d] | 1.75 (1.34–2.30) [d] | 1.63 (1.20–2.22) [c] | 1.81 (1.50–2.19) [d] | 1.55 (1.26–1.90) [d] |
| Health-related quality of life (Ref = High) | | | | | | |
| Moderate/low | 3.08 (2.25–4.23) [d] | 2.43 (1.78–3.32) [d] | 2.26 (1.44–3.54) [d] | 1.87 (1.19–2.93) [c] | 3.0 (2.07–4.36) [d] | 2.26 (1.55–3.31) [d] |
| Experience of pregnancy danger signs [e] (Ref = No**)** | | | | | | |
| Yes | 2.83 (2.44–3.28) [d] | 2.60 (2.22–3.04 [d] | 2.57 (2.02–3.26) [d] | 2.31(1.79–2.99) [d] | 2.88 (2.43–3.43) [d] | 2.67 (2.22–3.20) [d] |
| Experience of pregnancy complications [f] **(Ref = No)** | | | | | | |
| Yes | 2.06 (1.71–2.48) [d] | 1.87 (1.53–2.29) [d] | 2.89 (2.18–3.83) [d] | 2.67 (1.97–3.60) [d] | 1.99 (1.60–2.48) [d] | 1.82 (1.43–2.31) [d] |
| **Quasi-likelihood information criterion unbiasedness (QICu)** | **4373.64** | | **1563.3** | | **3051.8** | |

**Model I:** Pregnant women with at least two measures of depressive symptoms and followed up to six time-point measurements, regardless of trimester level.
**Model II**: First-trimester women who had at least two measures of depressive symptoms and were followed up to six measures of depressive symptoms.
**Model III**: Pregnant women with four completed measures of depressive symptoms, regardless of trimester level.
**Ref:** Reference group. [a] P-value < 0.25, [b] < 0.05, [c] < 0.01, [d] < 0.001. [e] Including severe or persistent headache, vaginal bleeding, fever, severe abdominal pain, difficulty breathing, convulsion or seizure, loss of consciousness, blurring of vision, and noticing the baby completely stopped moving. [f] Including any of preeclampsia, hyperemesis gravidarum, oligo/polyhydramnios, and RH-isoimmunization.
