# Supplementary material for: Magnitude, trajectory and determinants of antenatal depressive symptoms in Adama Town and East Shewa Zone, Ethiopia: evidence from a prospective longitudinal eCohort survey
Source: BMJ Open. 2026 Oct 7;16(10):e120181. doi: 10.1136/bmjopen-2026-120181 (PMC13646975; doi:10.1136/bmjopen-2026-120181)
Supplement: Supplementary file 6 [file bmjopen-16-10-s006.pdf]

**Supplementary Table 6**: Determinants of antenatal depressive symptoms using a cutoff point PHQ-9 ≥ 5 vs PHQ-9 ≥10

| Variable | Depressive symptoms [e] | | Model I | | Model II | |
|---|---|---|---|---|---|---|
| | Experienced (%) | Not Experienced (%) | cOR, 95%CI | aOR, 95%CI | cOR, 95%CI | aOR, 95%CI |
| Educational status | | | | | | |
| No education or some primary | 33.61 | 66.39 | 1.24 (0.95, 1.62) [a] | 0.99 (0.74 - 1.34) | 1.38 (0.91-2.09) [a] | 1.10 (0.70-1.74) |
| Complete primary or some secondary | 28.44 | 71.56 | 0.99 (.76 - 1.29) | 0.81 (0.62 - 1.06) | 0.98 (0.64-1.50) | 0.82 (0.53-1.26) |
| Complete secondary or higher | 28.69 | 71.31 | 1 | 1 | 1 | 1 |
| Marital status | | | | | | |
| Currently married | 30.04 | 69.96 | 0.45 (0.23 - 0.87) [b] | 0.53 (0.28 - 0.98) [b] | 0.37 (0.16-0.86) [b] | 0.48 (0.19-1.22) |
| Currently not married | 52.05 | 47.95 | 1 | 1 | 1 | 1 |
| Wealth index | | | | | | |
| Poorest | 33.02 | 66.98 | 1.29 (1.01 - 1.64) [b] | 1.13 (0.86 - 1.48) | 1.24 (0.86-1.81) [a] | 0.99 (0.64-1.52) |
| Middle | 30.42 | 69.58 | 1.13 (.88 - 1.44) | 1.01 (0.79 - 1.29) | 1.18 (0.81-1.71) | 1.02 (0.68-1.50) |
| Richest | 28.16 | 71.84 | 1 | 1 | 1 | 1 |
| Religion | | | | | | |
| Christian | 32.24 | 67.76 | 1.71 (0.99 - 1.96) [a] | 1.31 (0.71 - 2.41) | 5.42 (1.78-16.51) [c] | 3.76 (1.19-11.91) [b] |
| Muslim | 26.36 | 73.64 | 1.25 (0.70 - 2.22) | 0.93 (0.49 - 1.74) | 3.45 (1.09-10.91) [b] | 2.46 (0.75-8.12) |
| Others [f] | 22.63 | 77.37 | 1 | 1 | 1 | 1 |
| Gestational age | | | | | | |
| First trimester | 37.04 | 62.96 | 1.25 (0.96 - 1.63) [a] | 1.45 (1.09 - 1.93) [b] | 1.22 (0.83-1.78) | 1.26 (0.85-1.86) |
| Second trimester | 28.96 | 71.04 | 0.91 (0.81 - 1.03) [a] | 0.92 (0.80 - 1.06) | 0.86 (0.70-1.04) [a] | 0.85 (0.69-1.06) |
| Third trimester | 31 | 69 | 1 | 1 | 1 | 1 |
| Pregnancy Intention | | | | | | |
| Unintended | 35.41 | 64.59 | 1.36 (1.09 - 1.68) [c] | 1.29 (1.03 -1.62) [b] | 1.38 (0.99-1.89) [b] | 1.29 (0.90-1.85) |
| Intended | 28.8 | 71.2 | 1 | 1 | 1 | 1 |
| Experience of intimate partner violence | | | | | | |
| Yes | 56.15 | 43.85 | 3.09 (2.11 - 4.53) [d] | 2.43 (1.67 - 3.56) [d] | 3.11 (1.99-4.86) [d] | 1.96 (1.22-3.15) [c] |
| No | 28.72 | 72.28 | 1 | 1 | | 1 |
| Self-reported health status | | | | | | |
| Poor, fair, good | 35.89 | 64.11 | 1.88 (1.61 - 2.19) [d] | 1.66 (1.40 - 1.97) [d] | 2.54 (1.94-3.34) [d] | 2.11 (1.57-2.84) [d] |
| Very good, excellent | 20.88 | 79.12 | 1 | 1 | | 1 |
| Health-related quality of life | | | | | | |
| Moderate/low | 53.5 | 46.5 | 3.08 (2.25 - 4.23) [d] | 2.43 (1.78 - 3.32) [d] | 2.82 (1.90-4.19) [d] | 2.25 (1.53-3.33) [d] |
| High | 28.28 | 71.72 | 1 | 1 | | 1 |
| Experience of pregnancy danger signs [g] | | | | | | |
| At least one | 46.29 | 53.71 | 2.83 (2.44 - 3.28) [d] | 2.60 (2.22 - 3.04 [d] | 3.33 (2.62-4.25) [d] | 2.75 (2.13-3.54) [d] |
| No | 16.94 | 83.06 | 1 | 1 | | 1 |
| Experience of obstetric problems [h] | | | | | | |
| At least one | 49.83 | 50.17 | 2.06 (1.71 - 2.48) [d] | 1.87 (1.53 - 2.29) [d] | 2.43 (1.89-3.12) [d] | 2.04 (1.58-2.64) [d] |
| No | 27.2 | 72.8 | 1 | 1 | | 1 |

**Model I**: Defining antenatal depressive symptoms with PHQ-9 ≥5. **Model II**: Defining antenatal depressive symptoms with PHQ-9 ≥10 .1: Reference group, [a] P-value < 0.25, [b] < 0.05, [c] < 0.01, [d] < 0.001, [e] Values in the table are row percentages. [f] Including Adventist and traditional. g Includes severe or persistent headache, vaginal bleeding, fever, severe abdominal pain, difficulty breathing, convulsion or seizure, loss of consciousness, blurring of vision, and noticing the baby completely stopped moving. [h] Including any of preeclampsia, hyperemesis gravidarum, oligo/polyhydramnios, and RH-isoimmunization
