# Supplementary material for: Magnitude, trajectory and determinants of antenatal depressive symptoms in Adama Town and East Shewa Zone, Ethiopia: evidence from a prospective longitudinal eCohort survey
Source: BMJ Open. 2026 Oct 7;16(10):e120181. doi: 10.1136/bmjopen-2026-120181 (PMC13646975; doi:10.1136/bmjopen-2026-120181)
Supplement: Supplementary file 7 [file bmjopen-2026-120181.reviewer_comments.pdf]

PEER REVIEW HISTORY

BMJ Open publishes all reviews undertaken for accepted manuscripts. Reviewers are asked to complete a checklist review form and are provided with free text boxes to elaborate on their assessment. These free text comments are reproduced below.

ARTICLE DETAILS

Title (Provisional)

Magnitude, trajectory, and determinants of antenatal depressive symptoms in Adama Town and East Shewa Zone, Ethiopia: Evidence from a prospective longitudinal eCohort survey

Authors

Wondim, Gebeyaw Molla; Mekonnen, Wubegzier; Arsenault, Catherine; Mebratie, Anagaw Derseh; Kruk, Margaret; Haile Mariam, Damen

---

VERSION 1 - REVIEW

**Reviewer**	**1**
**Name**	**Mekonnen , Alemayehu Gonie**
**Affiliation**	**The University of Queensland School of Public Health**
**Date**	**12-Jun-2026**
**COI**	**None**

Manuscript ID: bmjopen-2026-120181 - Original research

Manuscript Title: **Magnitude, trajectory, and determinants of antenatal depressive symptoms in Adama Town and East Shewa Zone, Ethiopia: Evidence from a prospective longitudinal eCohort survey**

Comment for Authors

Thank you for the opportunity to review this manuscript, which used data from the eCohort study. The authors employed group-based trajectory modelling to assess patterns of depressive symptoms over time and generalized estimating equations to account for repeated measures.

I found the study highly relevant and timely. The finding that early pregnancy may represent a particularly vulnerable period for depressive symptoms is important, given the potential consequences for maternal health and pregnancy outcomes. These findings underscore the

need to strengthen holistic antenatal care that addresses both physical and psychological wellbeing.

The study also identified several factors associated with an increased risk of antenatal depressive symptoms, including unintended pregnancy, intimate partner violence, pregnancy complications, poor self-rated health, and reduced quality of life. These are important public health concerns that warrant further attention in maternal healthcare services and practice. Given the significance of the topic and the contribution of the study, I have some comments and suggestions that the authors may wish to consider before publication.

1. The abstract is well structured and clearly presented. However, in line 57, I suggest replacing "followed to the end of pregnancy" with "followed throughout their pregnancy", as this wording reads more smoothly.
2. In line 69, I recommend revising "pregnancy danger signs" and "pregnancy complications" to more specific and measurable terms. For clarity, these could be written as "experiencing at least one pregnancy danger sign" and "having a history of at least one obstetric complication", respectively. This wording better reflects the exposure definition and improves the interpretability of the findings.
3. The conclusion was derived from the main findings, but "Women's psychosocial, pregnancy-related problems, self-rated health status, and quality of life were positively associated with antenatal depressive symptoms" is too broad. I would replace this with "The high burden of antenatal depressive symptoms and their association with preventable social and pregnancy-related factors underscore the need for comprehensive antenatal care that incorporates mental health screening, psychosocial support, and interventions addressing intimate partner violence and pregnancy-related complications."
4. Line 119: "(economic difficulties, unfavourable marital conditions, and food insecurity)" are unnecessary details. I recommend revising this or maybe deleting it.
5. Line 239: "Data cleaning and analysis were conducted in Excel and Stata version 18 software" may be deleted.
6. Check the font size for tables 1, 2, 3, 4,…, and their descriptions.
7. Table 4: can be a supplementary file.

**Reviewer**	**2**
**Name**	**Pariante, Carmine**
**Affiliation**	**King's College London, Psychological Medicine**
**Date**	**28-Jul-2026**
**COI**	**None**

Thanks for such an interesting paper. it would further benefit from a discussion on the potential role of COVID in such a high prevalence, considering that the study was conducted shortly after the pandemic. Perhaps a historical comparison with studies conducted before COVID in similar areas or geographical/cultural settings could help shed ligh on this issue, or any data collected on covid infection in the participants.

**Reviewer**	**3**
**Name**	**Jayawardane, Asanka**
**Affiliation**	**University of Colombo Faculty of Medicine, Obstetrics and Gynecology**
**Date**	**30-Jul-2026**
**COI**	**None**

Thank you for the opportunity to review this manuscript of a methodologically ambitious study that could make a valuable contribution to the perinatal mental health literature in Ethiopia and in similar settings. I read the evaluation on antenatal depressive symptoms with great interest. The topic is timely and with key clinical, health care delivery policy and economic importance- and hardly explored previously.

The use of a longitudinal design (with monthly follow-up -appropriate for trajectory analysis) and advanced trajectory modelling addresses a genuine gap in the literature, and the large sample size ((n=933) with adequate power for GBTM and GE) from a well-described cohort is a valued asset.

I believe the study results merit dissemination and publication for peer evaluation and has some suggestions for clarity.

I must commend the authors for their robust data quality control (REDCap, audio recording, female data collectors, pilot testing) which is a delight to see.

The use of locally validated PHQ-9 tool which has good sensitivity/specificity is a strength. However the cut off of 5 means the study measures any depressive symptoms, not clinical depression and may explain the high prevalence (30.5%).

In the discussion please justify this cut-off. Also consider a sensitivity analysis using the ≥10 cut-off (moderate symptoms) to show whether the trajectory groups and determinants remain consistent. If not, the conclusions about "one-third" needs to be interpreted with caution.

The abstract states symptoms were higher "at first trimester, both at baseline and follow-up." If follow-up occurred at monthly intervals (women would progress to second/third trimester) this may indicate incorrect terminology. Please clarify the exact gestational weeks for each of the 7 measurement points. Specify whether women who entered in the first trimester were followed through to term or if new women entered at later trimesters.

Please include the results of the attrition analysis in the results section or as a supplement

Limitations- Acknowledge the sever attrition.

Only 85 of 933 participants completed all 7 rounds. Presenting the "cumulative retention" of 59.6% indicates that women ended their pregnancy and exited the study (which is expected), but the study does not distinguish between women who dropped out vs. women who completed their pregnancy and were no longer eligible. Please consider adding a competing risks analysis or clearly separate "lost to follow-up" from "pregnancy ended," and compare baseline characteristics of completers vs. non-completers.

The methods mention that only participants with at least two measurements were included, but there is no description of how missing data within the PHQ-9 (item level) were handled.

The conclusion states "one in ten demonstrated steadily increased depressive symptoms over time." However, the 11.6% group may not be clinically significant if they only had mild symptoms (PHQ-9 ≥5). Consider amending the conclusion by specifying this was for mild-to-moderate symptoms and acknowledging that clinical depression (PHQ-9 ≥10) may have a different trajectory.

VERSION 1 - AUTHOR RESPONSE

- **Response to Reviewer #1 (Alemayehu Gonie Mekonnen)**

Comment 1: The abstract is well structured and clearly presented. However, in line 57, I suggest replacing “followed to the end of pregnancy” with “followed throughout their pregnancy", as this wording reads more smoothly.

Authors’ response: We thank you for suggesting this and have revised accordingly, **line number 57**

Comment 2: In line 69, I recommend revising “pregnancy danger signs” and “pregnancy complications” to more specific and measurable terms. For clarity, these could be written as “experiencing at least one pregnancy danger sign” and “having a history of at least one

obstetric complication", respectively. This wording better reflects the exposure definition and improves the interpretability of the findings.

Authors' response: We thank you for the suggestion. We revised the abstracts as per the suggestion, **line numbers 70- 71**

Comment 3. The conclusion was derived from the main findings, but "Women's psychosocial, pregnancy-related problems, self-rated health status, and quality of life were positively associated with antenatal depressive symptoms" is too broad. I would replace this with "The high burden of antenatal depressive symptoms and their association with preventable social and pregnancy-related factors underscore the need for comprehensive antenatal care that incorporates mental health screening, psychosocial support, and interventions addressing intimate partner violence and pregnancy-related complications."

Authors' response: thank you for this constructive suggestion for making it more streamlined; we have revised the conclusions on **page 4, line numbers 76-80**.

Comment 4: Line 119: "(economic difficulties, unfavorable marital conditions, and food insecurity)" are unnecessary details. I recommend revising this or maybe deleting it.

Authors' response: Thank you for the comments and suggestions as well. We revised the paragraph considering the comments on **page 6, line numbers 120-121**

Comment 5: Line 239: "Data cleaning and analysis were conducted in Excel and Stata version 18 software" may be deleted.

Authors' response: We thank you for the thoughtful comments. However, retaining the details of our data cleaning and analysis is important for ensuring methodological transparency. Therefore, we have opted to keep this sentence as written on page 11, line number 240

Comment 6. Check the font size for Tables 1, 2, 3, 4,…, and their descriptions. Table 4: can be a supplementary file.

Authors' response: Thank you for the comments, and we revised the font size of all tables by making the standard font size (12 font size).

- **Response to Reviewer #2 (Dr. Carmine Pariante)**

Comments 1. Thanks for such an interesting paper. It would further benefit from a discussion on the potential role of COVID in such a high prevalence, considering that the study was conducted shortly after the pandemic. Perhaps a historical comparison with studies conducted

before COVID in similar areas or geographical/cultural settings could help shed light on this issue, or any data collected on COVID infection in the participants.

Authors' response. We thank you for this important feedback and comments. As you mention, our study was conducted during the post-pandemic COVID-19 period, and the burden of antenatal depressive symptoms is relatively higher in the current study. We agree that post-COVID-19 may have a direct and indirect impact on antenatal depressive symptoms. However, this high burden of antenatal depressive symptoms was comparable with studies conducted after COVID-19 in similar district (*Chala, A. et al. (2026)): https://link.springer.com/article/10.1186/s12978-026-02346-5), reference number 58* and before COVID-19 *(Tiki, T., Taye, K. and Duko, B. (2020): https://doi.org/10.1186/s12991-020-00275-6), reference number 57.* Consequently, our study findings were discussed with studies conducted before COVID-19 and after COVID-19, and the appropriate articles were cited on **page 23, line numbers 448-449.** Despite this, we acknowledged the lack of COVID-19 data, specifically whether the women had a history of COVID-19 or not.

- **Response to Reviewer #3 (Dr. Asanka Jayawardane)**

Comment 1. The use of a locally validated PHQ-9 tool, which has good sensitivity/specificity, is a strength. However, the cut-off of 5 means the study measures any depressive symptoms, not clinical depression, and may explain the high prevalence (30.5%). In the discussion, please justify this cut-off.

Authors' response: We thank you for this critical comment. We agree that a PHQ-9≥5 indicates the presence of depressive symptoms and is most used as screening for depressive symptoms rather than clinical depression. Consequently, we used this cut-off point due to the public health importance of antenatal depressive symptoms, including for promoting the importance of depressive symptoms screening across the women's continuum of care. This is because a significant number of women are undiagnosed for depression because of poor screening of depressive symptoms during pregnancy and its similarity with pregnancy-related symptoms. As a result, defining depressive symptoms broadly is very important for pregnant women to enhance mental health services across the continuum of care. Additionally, the PHQ-9 was validated in a similar setting among pregnant women with a cut-off point ≥ 5, which is a highly prevalent and essential cut-off point for low-primary health care services. Consequently, we justified the reason that we used a cut-off point ≥5 in the discussion section **on page 24, numbers 459-463**.

Comment 2. Consider a sensitivity analysis using the ≥10 cut-off (moderate symptoms) to show whether the trajectory groups and determinants remain consistent. If not, the conclusions about "one-third" need to be interpreted with caution.

Authors' response: We thank you for these thoughtful comments. We conducted a sensitivity analysis considering PHQ-9 ≥10. The results at both cutoff points were similar to the original result for a PHQ-9 ≥5. However, only two variables included, pregnancy intention and gestational age, did not show a significant association with antenatal depressive symptoms. Consequently, our study findings were robust, while the interpretation of these two variables should be approached with caution. According to the comment, we revised the manuscript by incorporating a sensitivity analysis on **page 13, line numbers 290-294, and page 20, line numbers 420-430**

Comment 3. The abstract states symptoms were higher "at first trimester, both at baseline and follow-up." If follow-up occurred at monthly intervals (women would progress to second/third trimester), this may indicate incorrect terminology. Please clarify the exact gestational weeks for each of the 7 measurement points. Specify whether women who entered in the first trimester were followed through to term or if new women entered at later trimesters.

Authors' response: Thank you for this important comment to clarify the ambiguous terminology. In this study, the pregnant women were enrolled irrespective of their gestational age in their first ANC and followed through until delivery (employing a prospective closed cohort study design, no women enrolled during follow-up). Women were recruited at their first, second, and third trimesters at baseline and were followed monthly until delivery. Hence, no fixed gestation age occurs across the measurement points. As a result, the seven measurement points indicate the individual assessment time, which was conducted every month rather than the fixed gestational age to assess their depressive symptoms. Women's gestational age at visit 2 and subsequent visits was dependent on the women's gestational age at baseline (Visit 1). Consequently, we clarified that the first trimester at baseline and the follow-up period: first trimester at baseline is those women whose gestational age is less than 13 weeks, while first trimester at follow-up indicates those women who were recruited at baseline and their gestational age was still less than 13 weeks at the second visit/assessment. According to this recruitment and follow-up process, we revised the abstract that made an ambiguous statement **on page 3, line numbers 65-66.**

Comment 4. Please include the results of the attrition analysis in the results section or as a supplement

Authors' response: Thank you for your feedback. We conducted the attrition analysis by presenting the participant flow diagram across each recruitment point that showed how many participants were evaluated for depressive symptoms and other pregnancy-related problems, including danger signs and pregnancy complications, and how many of them were absent in each measurement point. As presented on page 14 in the participants' flow diagram, all participants were evaluated for depressive symptoms in the first two rounds of the survey, while 91.5%, 78%, and 54.2% were evaluated in the third, fourth, and fifth rounds, respectively. The primary reasons for dropping out of the study were the end of pregnancy or not further eligible for assessing antenatal depressive symptoms at the measurement point (82.4%) and inability to continue follow-up (lost follow-up) (8.5%). Hence, the attrition result table is presented (**Table 1)** and revised on **page 14, line numbers 311-316**. Furthermore, the attrition bias analysis is also presented as supplementary Table 3 on **page 18, line numbers 384-390**

Comment 5. Limitations- Acknowledge the severe attrition. Only 85 of 933 participants completed all 7 rounds. Presenting the "cumulative retention" of 59.6% indicates that women ended their pregnancy and exited the study (which is expected), but the study does not distinguish between women who dropped out vs. women who completed their pregnancy and were no longer eligible.

Authors' response: We thank you for your critical and thoughtful comment. We acknowledge the presence of high attrition as a limitation. This high attrition was due to two main reasons, including no longer being eligible to assess antenatal depressive symptoms because of the pregnancy outcome (delivery/ stillbirth/abortion) and lost follow-up. Consequently, 79 (8.5%) of pregnant women dropped out, and 769 (82.4%) completed their pregnancy, including delivered alive newborns (745 women) and abortion/stillbirth (24 women) before completing all seven rounds. All these important comments are presented and revised in the study flow table (attrition result) **on page 14, line numbers 312-316.**

Comment 6. Please consider adding a competing risks analysis or clearly separating "lost to follow-up" from "pregnancy ended," and compare baseline characteristics of completers vs. non-completers.

Authors' response: We thank you for your critical comment. We agree that treating all non-completers as one group introduces a selection bias. To address this, we categorized all participants by follow-up status, including those who completed follow-up, those who did not complete the survey due to pregnancy outcome (i.e., delivered a live newborn, stillbirth, or abortion), and those who were assessed up to six or seven rounds of evaluation. Consequently, we conducted a fine-gray competing risk regression treating true dropouts from follow-up and those who dropped out due to delivery/abortion/miscarriage as competing events. Using this approach, we conducted a multivariate subdistribution hazard ratio (sHR) analysis to identify predictors of true lost follow-up at six and seven measurement points.

After adjusting for all baseline covariates, the model showed that participants who experienced depressive symptoms at baseline, were currently married, resided in a rural area, were formerly employed, and reported a high HRQoL had a significantly lower risk of lost follow-up (higher retention). Conversely, younger age (< 20 years and between 20-34 years), poor wealth index, first trimester enrolment, self-reported poor health status, and the presence of at least one obstetric complication increased the risk of lost follow-up over the longitudinal follow-up (**Table S3**).

Further, we compared participants who completed the follow-up and those who did not complete the six-round survey: the results demonstrated significant baseline differences between the two groups across their baseline characteristics (**Table S2**). As a result, we emphasized that the study findings should be interpreted cautiously due to high attrition. We have incorporated these comments and revised them into the manuscript **on pages 12, line numbers 267-275** (Method section) **and Page 19, lines 391-401 (**Results section).

Comment 7. The methods mention that only participants with at least two measurements were included, but there is no description of how missing data within the PHQ-9 (item level) were handled.

Authors' response: Thank you for your thoughtful comment. We used a complete case analysis, considering the missing data completely at random assumption. However, we used mean imputation for PHQ-9 item-specific missing data across each measurement point. Consequently, we revised and updated the method of the data management section on **page 11, line numbers 241-243.**

Comment 8. The conclusion states "one in ten demonstrated steadily increased depressive symptoms over time." However, the 11.6% group may not be clinically significant if they only had mild symptoms (PHQ-9 ≥5). Consider amending the conclusion by specifying this was for mild-to-moderate symptoms and acknowledging that clinical depression (PHQ-9 ≥10) may have a different trajectory.

Authors' response: We thank you for the thoughtful comments as well. We agree that PHQ-9 is mainly used for screening depressive symptoms, rather than a clinical diagnosis of depression, and we defined women's experience of depressive symptoms with a PHQ-9 ≥ 5. Consequently, 11.6% of pregnant women experienced a trajectory of mild-to-moderate depressive symptoms that steadily increased over time, and we revised the conclusion by incorporating the comments **on page 27, line number 539/41**

VERSION 2 - REVIEW

**Reviewer**	**3**
**Name**	**Jayawardane, Asanka**
**Affiliation**	**University of Colombo Faculty of Medicine, Obstetrics and Gynecology**
**Date**	**31-Aug-2026**
**COI**	

Thank you. I read the resubmitted document with great interest. I believe the article is much improved and now ready for publication. My following comments are fully addressed.

Handle missing data within PHQ-9 (item level)- Methods (Lines 241–243)

Detail the baseline vs. follow-up attrition analysis- Results (Lines 384–390) & Supp. Table 2

Perform a competing risks analysis for dropouts- Methods (Lines 267–275), Results (Lines 391–401) & Supp. Table 3

Conduct sensitivity analysis using a PHQ-9 ≥10 cut-off- Methods (Lines 292–294), Results (Lines 426–430) & Supp. Table 6

Justify the PHQ-9 ≥5 cut-off in the Discussion- Discussion (Lines 441–446)

Acknowledge severe attrition as a limitation- Strengths/Limitations (Lines 89–90), Discussion (Lines 413–419, 461–463)

Amend Conclusion text to specify "mild-to-moderate"- Abstract (Line 67), Conclusion (Lines 75–76, 540–542)

Further suggestions

Clarification of exact gestation points at the 7 measurement points-Please consider a statement clarifying explicitly in the Methods or Results how the longitudinal alignment handles varying gestational entry ages

Good Luck!

VERSION 2 - AUTHOR RESPONSE

Reviewer #3 comments

Comment #1: Clarification of exact gestational points across the seven measurement points (Further suggestions). Please consider a statement clarifying explicitly in the Methods or Results how longitudinal alignment handles varying gestational entry ages.

Response*: Thank you for your important comments clarifying the women's gestational age across each measurement point. We have revised and added how the longitudinal alignment of gestational age at entry and follow-up period is handled across the seven measurement points* ***(page 10, lines 223-229****). Briefly, the baseline gestational age was determined based on the mother's self-reported LMP and the date of enrolment. For the follow-up period, the data were collected at fixed intervals (monthly). At each subsequent follow-up, the women's gestational age was determined by considering the baseline gestational age and the elapsed time from her baseline.*
